# Dimensionality in confirmatory factor analysis is not in the eye of the beholder: Ancillary bifactor statistical indices illuminate dimensionality and reliability

**DOI:** 10.1002/ijop.13266

**Published:** 2024-11-18

**Authors:** Tyrone B. Pretorius, Anita Padmanabhanunni

**Affiliations:** ^1^ Department of Psychology University of the Western Cape Cape Town South Africa

**Keywords:** Confirmatory factor analysis, Bifactor models, Model fit indices, Ancillary bifactor indices

## Abstract

This tutorial delves into dimensionality assessment within the context of psychological measurement instruments, particularly focusing on bifactor models. It underscores the imperative to move beyond traditional fit indices when evaluating factor structures while highlighting the significance of ancillary bifactor indices such as explained common variance, OmegaH and percentage of uncontaminated correlations in gaining a more comprehensive understanding of the interplay between general and specific group factors. The tutorial offers a step‐by‐step guide to leveraging the power of R software for confirmatory factor analysis and the acquisition of ancillary bifactor indices. Through practical case studies, it elucidates the potential pitfalls of exclusively relying on fit indices and advocates for a balanced, multifaceted approach to dimensionality assessment. By integrating fit measures and ancillary indices, researchers can draw more informed and nuanced conclusions about measurement instrument dimensionality, ultimately enhancing the precision of psychological assessment.

The psychometric properties of a measuring instrument are not embedded qualities but sample‐dependent. For example, concerning the reliability of scores produced by an instrument, Wilkinson and the American Psychological Association Task Force on Statistical Inference asserted that a test's reliability is not an intrinsic attribute but a characteristic of the scores derived from a specific test‐taking population (Wilkinson & APA Task Force, 1999). Hence, instruments may demonstrate disparate properties across distinct samples, highlighting the imperative for researchers to scrutinise the psychometric attributes of a scale prior to undertaking pertinent analyses. This investigation typically examines the reliability of scores produced by an instrument and the purported factor structure.

In instances where the authors of a questionnaire conceptualise the questionnaire as consisting of a total scale score as well as several subscale scores, researchers would test this conceptualisation through examining the following hypotheses using confirmatory factor analyses:

- The questionnaire consists only of a total scale—the one‐factor model.
- The questionnaire consists only of subscales that are related to each other—the correlated‐factor model.
- The questionnaire consists of a hierarchy of factors with the items of the questionnaire loading on a first order set of factors (subscales), and these first order set of factors grouped under a second order factor (total scale)—the second‐order factor model.
- All the items of the questionnaire are hypothesised to load on a general factor, while a subset of items loads on several specific group factors—the bifactor model

The one‐factor and the correlated‐factor models are regarded as nested models while the second‐order and bifactor models are not. A nested model is a simpler form of a more complex model. In this regard, if the factor correlations in the second‐order factor model were constrained to one, it would be the equivalent of the one‐factor model. The higher‐order factor and bifactor the models are direct rival models since both postulates the existence of a total scale as well as subscales and thus are often compared using CFA. In these model comparisons, several fit indices are used to assess the extent to which the model fits the data. While there is a wide range of fit indices, Kline (2015) suggested that the following indices should be used at a minimum: chi‐square (χ^2^), the root mean square error of approximation (RMSEA), the comparative fit index (CFI), the goodness‐of‐fit index (GFI) and the standardised root mean square residual (SRMR). A good fit is indicated by a non‐significant χ^2^ (although according to Jöreskog et al. (2016), this would indicate a perfect fit), an RMSEA less than .05, a CFI greater than .90, a GFI greater than .95, and an SRMR less than .08 (Byrne, 1994). A further useful index to include when comparing models is Akaike's information criterion (AIC); the model with the lowest AIC is considered the best‐fitting model.

The existing literature on CFA indicates that when a bifactor model of the structure of an instrument is compared to other models (e.g., a correlated two‐factor or higher order), the bifactor model most often emerges as the best‐fitting model. Therefore, researchers have claimed that the fit indices in CFA (e.g., a GFI or CFI) generally favour the bifactor model over other models (Bornovalova et al., 2020) as a result of overfitting due to the bifactor model having more parameters. In these instances, when the bifactor model emerges as the best model, researchers often conclude that the instrument under study is multidimensional based solely on fit indices.

However, apprehensions regarding the sole reliance on fit indices for deducing a scale's dimensionality have been articulated. McDonald (2010) suggested that an analysis that concludes with a globally fitted model, accompanied by global approximation indices, is incomplete and lacks informativeness. Morgan et al. (2015) similarly cautioned against the solitary reliance on approximate fit statistics, labelling it hazardous, while Rodriguez et al. (2016a) described such conclusions as an “overly simplistic conceptualisation of the dimensionality of psychological data” (p. 231).

Apart from these concerns, Bornovalova et al. (2020) highlight that there are various instances of model misfit, where a bifactor model can exhibit very good global fit, but the pattern of factor loadings is less than meaningful, for example small and negligible standardised coefficients (local model misfit) or standardised coefficients greater than 1. Models can also be manipulated to produce acceptable global fit through random post‐hoc modifications that is not grounded in theoretical foundations. Most statistical software packages provide modification indices, but Iacobucci (2009) refers to them as coming from the “statistics devil” as they seduce researchers into seeking better fit indices often resulting in nonsensical models that cannot be replicated in other samples. It should be noted that those taking advantage of post hoc model fit methods, typically never test that modified model with independent samples nor is that a baseline model used for future research.

If CFA identifies a bifactor structure as optimal, whether the specific group factors explain a sufficient amount of the variance in the items relative to the general factor is important to question. Pretorius (2021) indicated that three possible conclusions can be drawn when an instrument is identified by CFA fit indices as having a bifactor structure: (a) the specific group factors do not explain sufficient variance in the items, so the instrument should be considered unidimensional; (b) there is some evidence of multidimensionality albeit insufficient to rule out unidimensionality; or (c) the specific group factors explain a sufficient amount of variance to deem the instrument multidimensional.

The question of multidimensionality can only be resolved using ancillary bifactor indices (Rodriguez et al., 2016a). These indices include the explained common variance (ECV), omega hierarchical (OmegaH) and the percentage of uncontaminated correlations (PUC), the minimum indices needed to conclude the dimensionality of an instrument. ECV is the proportion of variance in all items explained by a factor—an ECV greater than .70 for the general factor generally indicates that the instrument under consideration is unidimensional (Rodriguez et al., 2016a). ECV is generally regarded as the critical index to conclude the data's dimensionality, indicating the relative strength of the general factor (Rodriguez et al., 2016b).

OmegaH is an estimate that represents the proportion of variance in unit‐weighted observed scores due to the general factor. For specific group factors, OmegaHS refers to the proportion of systematic variance in unit‐weighted scores accounted for by a specific group factor after the variance attributable to the general factor and other specific group factors is controlled. When OmegaH of the general factor is greater than .80, and OmegaHS is less than .50, the general factor is likely more reliable than the specific group factors (Schmitt et al., 2018).

PUC reflects the percentage of correlations between items accounted for by the general factor. A PUC value greater than .80 reflects a dominant general factor. Another index of interest is factor determinacy (FD), which is the correlation between factor scores and the factors. FD values greater than .90 reflect that factor scores are good estimates of individual differences on the factor. In addition to the above indices that relates to the dimensionality of an instrument, there are two indices that relate specifically to the reliability of scores produced by an instrument, namely Omega, which is a model‐based estimate of reliability similar to Cronbach's alpha, and the construct replicability coefficient H, an indication of the reliability or consistency of an optimally weighted latent factor and how well the observed variables (items) represent the underlying factor (Watkins & Canivez, 2022). In general, Omega values should be greater than .75, while H values greater than .80 indicate a well‐defined latent variable (Dueber, 2017).

This paper is primarily concerned with the implementation of ancillary bifactor indices following the fitting of a bifactor model. It should be noted, however, that there are similar procedures to decompose sources of variance that can be used with the higher‐order factor model (i.e., Schmid & Leiman, 1957). In addition, while ancillary bifactor indices can validate an instrument's hypothesised structure, it does not replace other forms of construct validity to ensure an instrument measures what it purports to measure.

## STRUCTURE DOES NOT EQUATE TO DIMENSIONALITY—TWO CASES IN POINT

This section uses two studies reported in the literature to demonstrate that inferences about dimensionality should not be based solely on CFA fit indices.^1^

## STUDY 1: VALIDATION OF THE CYBERCHONDRIA SEVERITY SCALE (CSS): REPLICATION AND EXTENSION WITH BIFACTOR MODELLING

**Figure 1:**
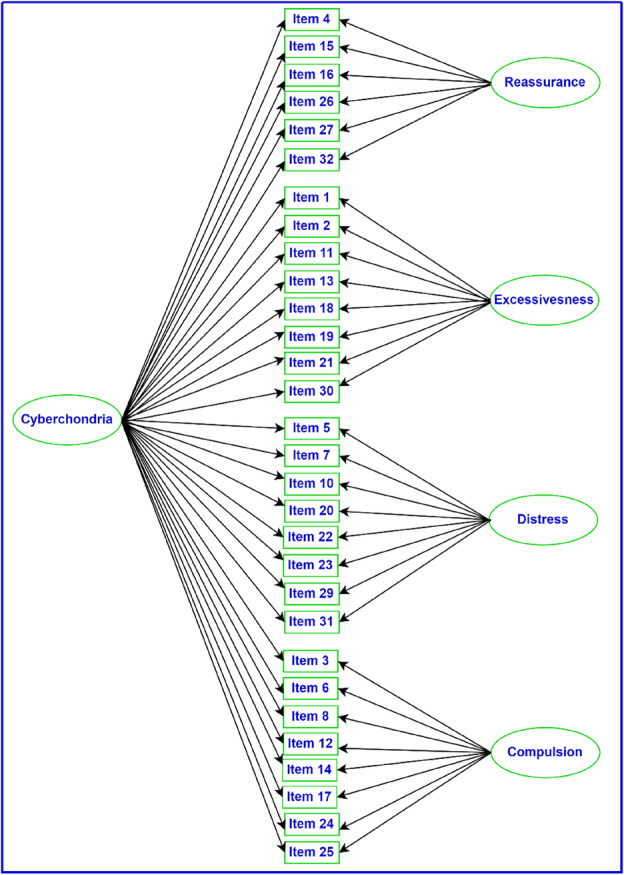
A bifactor model of the factor structure of the CSS. *Note*: Rectangles are observed variables; ellipses are latent variables.

As part of a larger study examining risk factors for cyberchondria, Norr et al. (2015) investigated several models of the factor structure of the CSS, including a bifactor model, in a convenience sample of 526 individuals with a mean age of 34.87 years (*SD* = 12.41). In the bifactor model, the CSS consists of a general factor (30 items) and four specific group factors: reassurance (six items), excessiveness (eight items), distress (eight items) and compulsion (eight items). The bifactor model is presented in Figure 1.

The authors found that the bifactor model provided the best fit to the data (χ^2^ = 1691.05, *p* < .001; CFI = .97; RMSEA .07) compared to a one‐factor, correlated (oblique) five‐factor, and partial second‐order factor. The study reported the standardised factor loadings, enabling the computation of ancillary bifactor indices to determine the relative strength of the general and specific group factors. As demonstrated later, these indices can be obtained in R (R Development Core Team, 2013), but there is also a freely available online Excel calculator (Dueber, 2017) at https://uknowledge.uky.edu/edp_tools/1/ that is simple and straightforward to use. A further advantage is that the calculator provides some references and defines every index. Screenshot 1 shows the spreadsheet where the standardised factor loadings reported in the study were entered.

**Screenshot 1:**
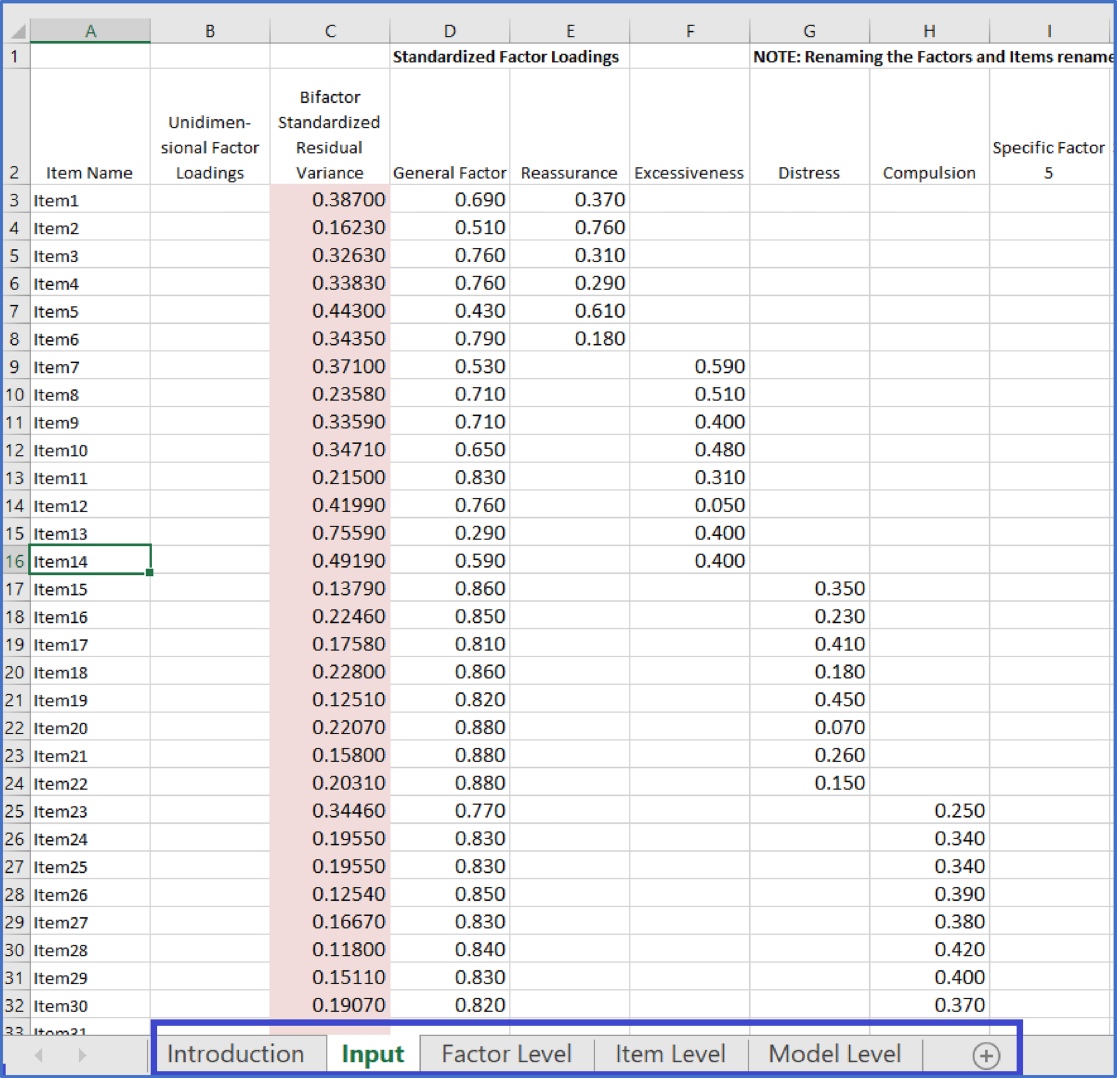
Input screen of the bifactor indices calculator for the CSS.

In the Excel spreadsheet, the blue box highlights five tabs:

- “Introduction” contains some directions and references.
- “Input” is the current sheet displayed where the standardised factor loadings are entered. Most statistical software packages provide factor analysis results in columns, so copying and pasting the factor loadings is advisable to avoid manual errors. The general factor loadings were entered in Column D, while specific group factor loadings were in Columns E to H. The names of the specific group factors were added in the headers of Columns E to H.
- “Factor Level” bifactor indices (e.g. ECV) were immediately calculated in the tab once the standardised factor loadings were entered into the “Input” tab, as discussed in Screenshot 3.
- “Item Level” is not used in the current tutorial but contains item‐ECV (IECV). It identifies items whose responses are accounted for by variation in the latent general dimension alone and is useful when researchers want to refine an instrument and develop a fairly unidimensional scale.
- “Model Level” contains the PUC value for the model, as shown in Screenshot 3.

The authors of this tutorial prefer the Dueber calculator over other software packages because every Excel sheet in the various tabs contains detailed commentary about the definition of indices and their interpretation. These are not included in the screenshots, but an example is shown in Screenshot 2.

**Screenshot 2:**
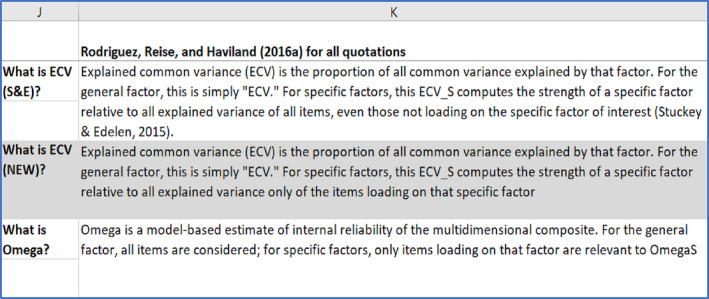
Example of commentary in the bifactor indices calculator.

The factor and model level indices in the “Factor Level” and “Model Level” tabs are shown in Screenshot 3.

**Screenshot 3:**
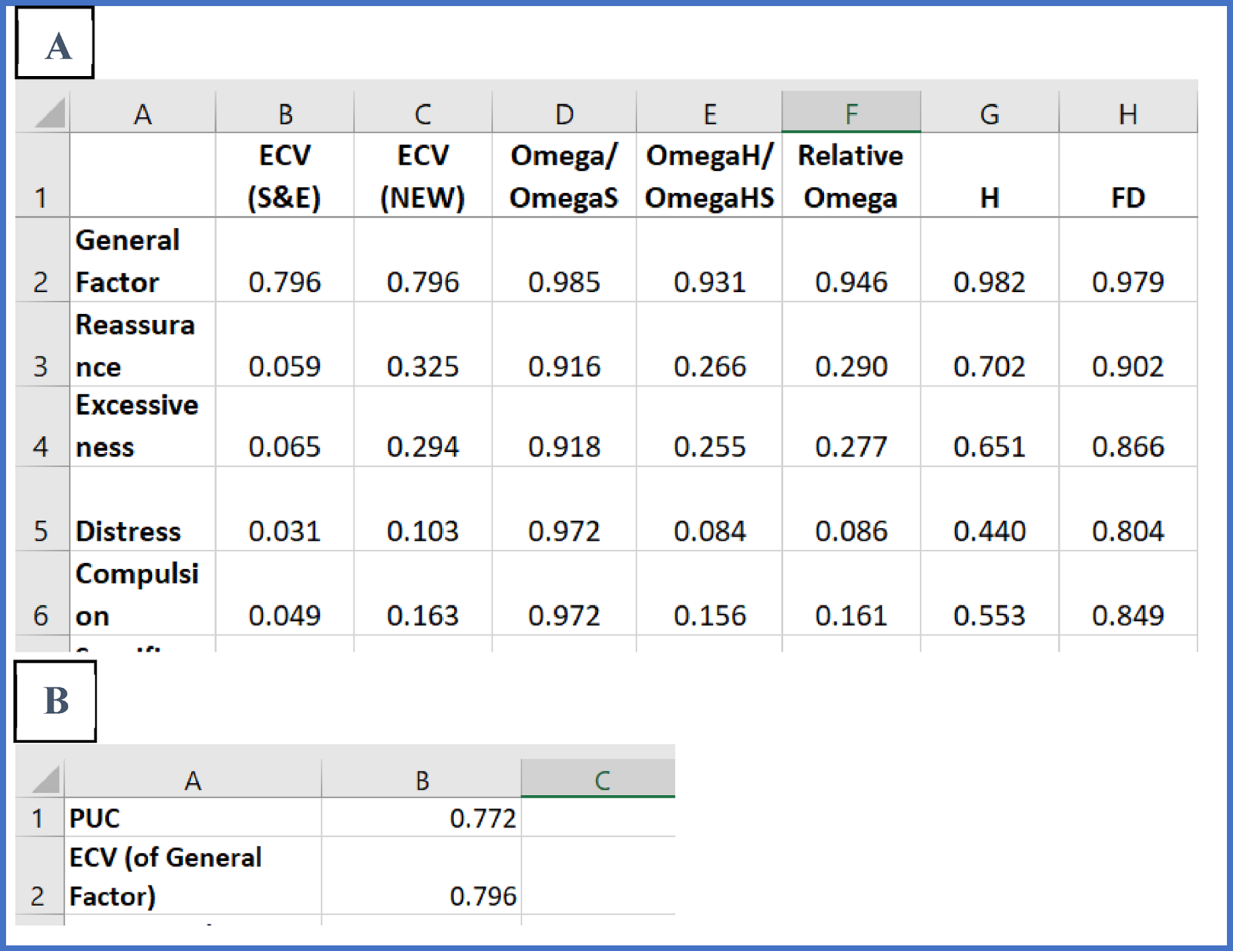
Factor and model level indices obtained with bifactor indices calculator for the CSS.

The indices for the general factor of the CSS reflected a strong, dominant factor. The general factor accounted for 79.6% of the item variance, while the four specific group factors accounted for only 20.4%. OmegaH of the general factor was above .80, while OmegaHS of the specific group factors were all below .50, indicating that the general factor was well represented by the item content while the specific group factors were not well indexed by their measured variables. The construct replicability coefficient (H) was greater than .80, and the FD was greater than .90. In sum, there was strong evidence that the CSS was essentially unidimensional. The four specific group factors did not seem to provide any additional valuable information over and above that provided by the general factor. If an unexpected finding of unidimensionality is obtained, when theory suggests or previous research provided evidence that an instrument is multidimensional, it is always recommended that different sources of evidence be explored to confirm unidimensionality. In this regard, the rule of thumb of “ratio‐of‐first‐to‐second‐eigenvalues‐greater‐than‐three criteria” provides a useful quick way to examine the importance of factors extracted through a factor analysis. This criterion suggests that when the ratio of the eigenvalue of the first factor extracted in a factor analysis to the eigenvalue of the second factor is greater than three the instrument should be regarded as unidimensional.

## STUDY 2: EXAMINING THE GENERALIZABILITY OF PROBLEM‐SOLVING APPRAISAL IN BLACK SOUTH AFRICANS

Heppner et al. (2002) compared a bifactor, a one‐factor, and a correlated (oblique) three‐factor model of the Problem Solving Inventory (PSI) in a sample of Black South African students (*n* = 447). The PSI consists of 32 items and measures individuals' appraisal of their problem‐solving abilities rather than their actual problem‐solving skills. The PSI consists of a total scale and three subscales: problem‐solving confidence (11 items), approach‐avoidance style (16 items), and personal control (five items). The Heppner study aimed to examine the generalisability of the PSI factor structure with Black South African students. Problem‐solving confidence refers to individuals' belief and trust in their problem‐solving ability. Approach‐avoidance style refers to the general propensity to avoid or approach problem‐solving tasks, while personal control reflects the extent to which individuals believe they control their emotions and behaviours during problem‐solving.

**Figure 2:**
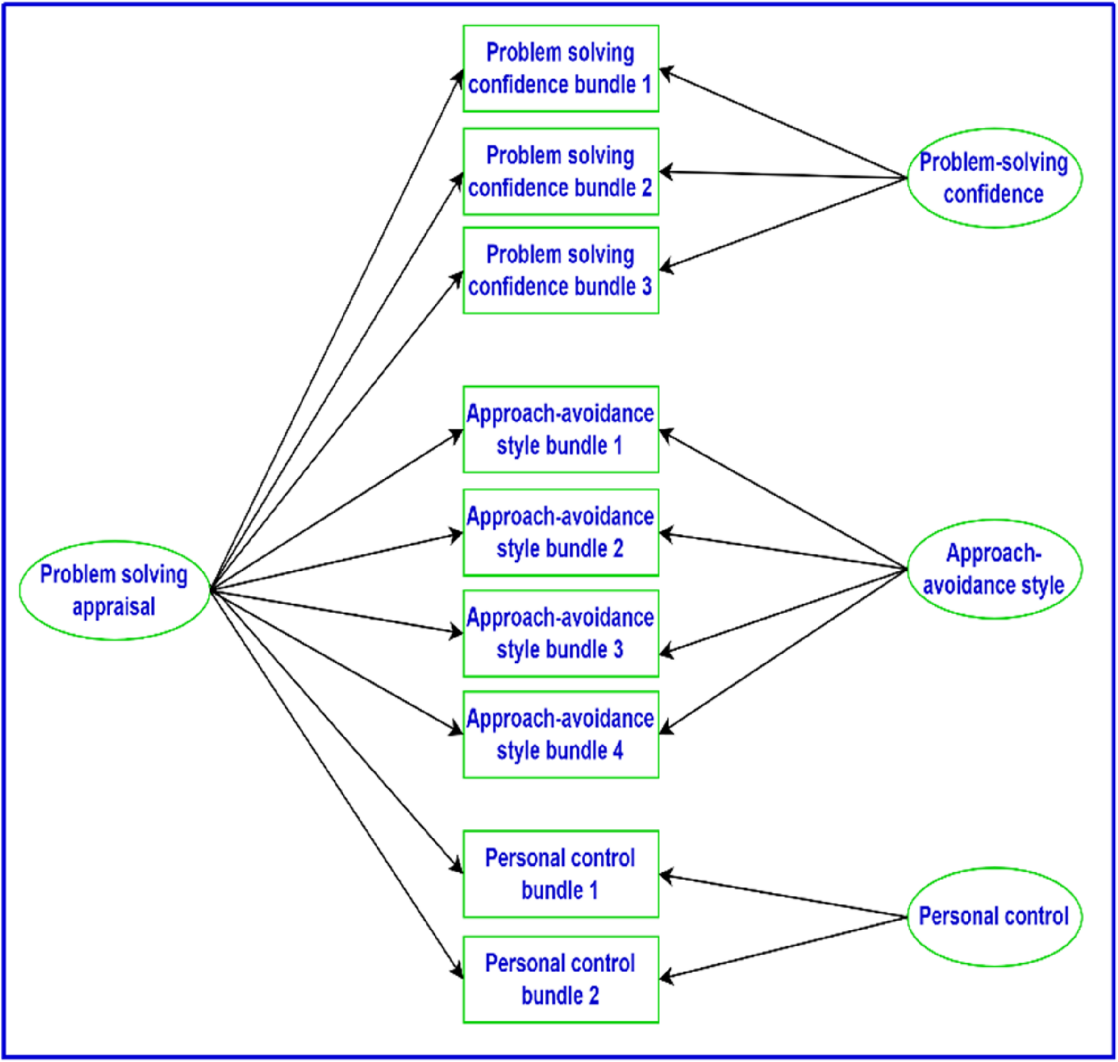
Bifactor model of the factor structure of the PSI. *Note*: Rectangles are observed variables; ellipses are latent variables.

The bifactor model of the PSI that Heppner and colleagues examined is presented in Figure 2. Notably, the study did not use the 32 items as indicators of the general and specific group factors, but rather used nine bundles of items to avoid estimating a large number of parameters and the potential distortion by idiosyncratic characteristics of individual items when fitting the model, as the authors indicated. Hence, the 32 PSI items were grouped into three bundles for problem‐solving confidence, four for approach avoidance style, and two for personal control. The study found the bifactor model to be a superior fit to the data (χ^2^ = 66.58, *p* < .001; CFI = .97; RMSEA .08), compared to a one‐factor and correlated (oblique) three‐factor model.

We used the Dueber calculator to further examine the dimensionality of the PSI. The “Input” sheet of the bifactor indices calculator for the PSI is shown in Screenshot 4.

**Screenshot 4:**
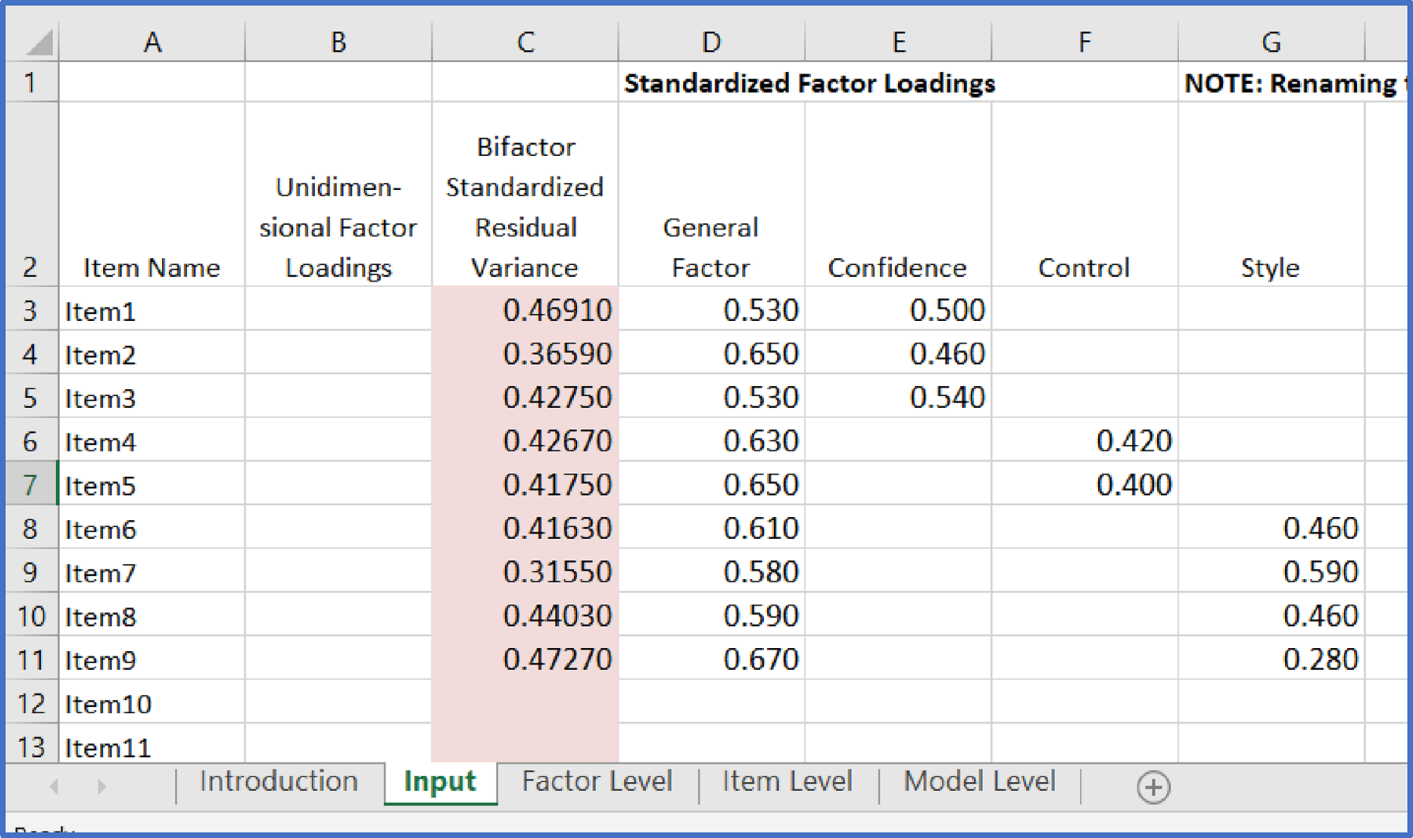
Input screen of the bifactor indices calculator for the PSI.

The factor and model level indices resulting from these standardised factor loadings are presented in Screenshot 5.

**Screenshot 5:**
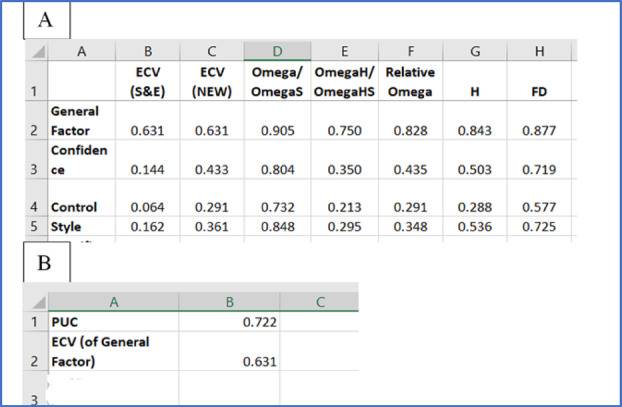
Factor and model level indices obtained with bifactor indices calculator for the PSI.

Screenshot 5 indicates that the general factor explained 63% of the variance (ECV), while the three specific group factors accounted for 37%. OmegaH of the general factor was below .80. These findings, together with the ECV, suggested that the PSI was multidimensional. However, Reise et al. (2013) suggested that the PUC, ECV, and OmegaH should be considered together when an instrument demonstrates some evidence of multidimensionality:

When PUC values are lower than .80, general ECV values greater than .60 and OmegaH > .70 [of the general factor] suggest that the presence of some multidimensionality is not severe enough to disqualify the interpretation of the instrument as primarily unidimensional. (p. 22)

In the case of the PSI, PUC values were lower than .80 (.72), the ECV was greater than .60 (.63), and OmegaH was greater than .70 (.75), thus indicating that the existing multidimensionality was insufficient, so the PSI should be regarded as unidimensional.

## AN ILLUSTRATIVE EXAMPLE USING R TO PERFORM A BIFACTOR ANALYSIS WITH ANCILLARY BIFACTOR INDICES: THE VACCINATION ATTITUDE SCALE^2^

This section demonstrates the use of R software to perform bifactor analyses and obtain ancillary bifactor analyses. The R script is attached as Appendix A. The Vaccination Attitudes Examination Scale (VAX) was developed as a multidimensional tool to assess general attitudes towards vaccination using 12 items. The VAX consists of a total score and four subscales: mistrust of vaccine benefits, worries about unforeseen future effects, concerns about commercial profiteering, and preference for natural immunity. The VAX is included in Appendix B. The data for the VAX used in R was obtained from students at a South African university (*n* = 322). The mean age of the sample was 26 years (*SD* = 10.2), and most of the sample was women (77%). The R script for specifying the bifactor model is shown in R Script 1.

**R Script 1:**
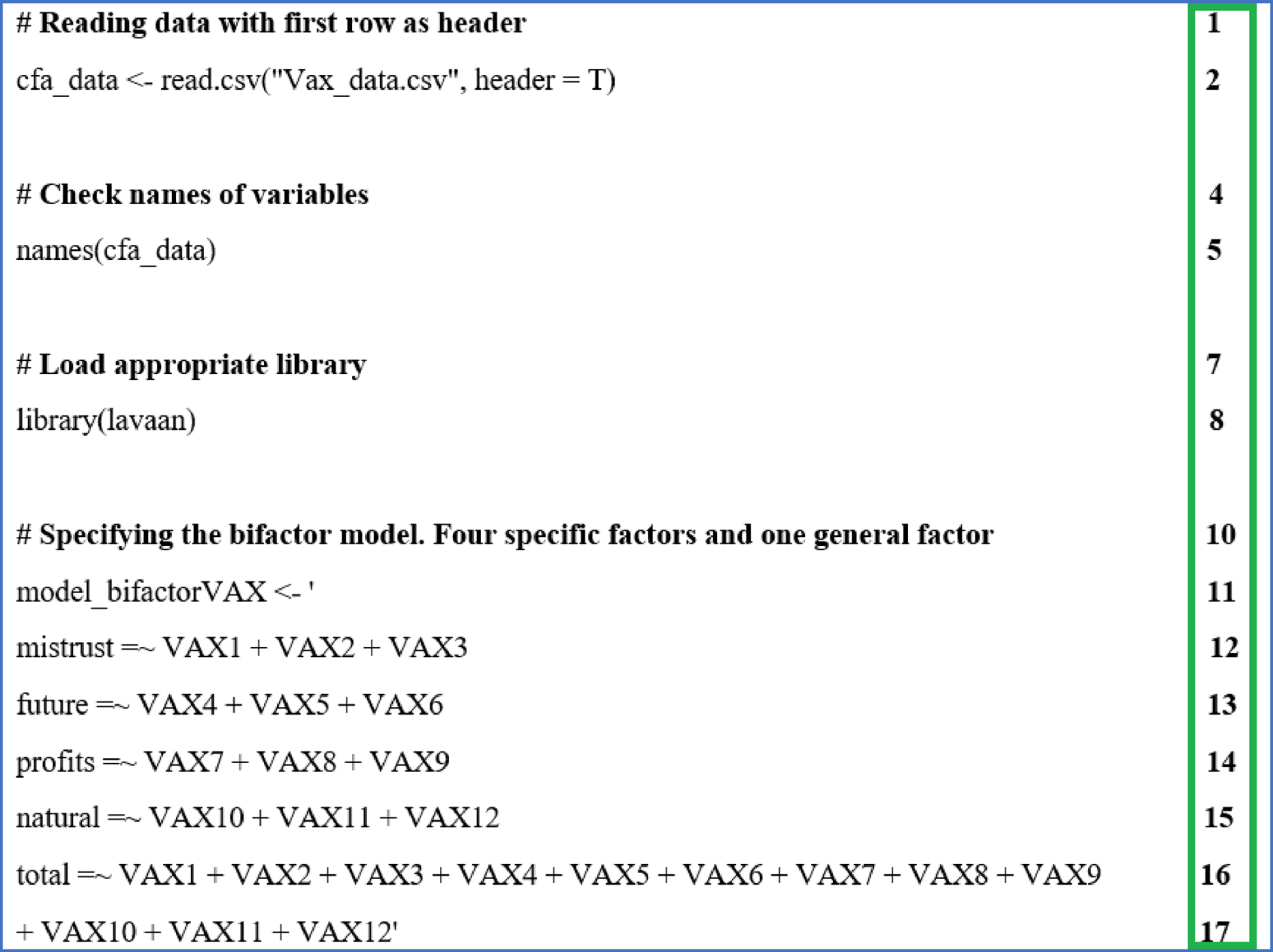
Specifying the bifactor model.

In R Script 1, the line numbers on the right in the green box are not part of the R script. They were only used in this manuscript for reference purposes.

- Line 2 is the command to read the data file. The data were saved as a comma‐separated value file in Excel. Thus, the command in line 2 created an R data file called cfa_data by reading the data in the CSV file called VAX_data. The statement header = T indicates that the first row in the CSV file contains the names of the variables. Using R Studio is strongly recommended, as the data file can be placed in the same folder as the project. Otherwise, in R, ensure the file path is correctly specified (e.g., c:/desktop/myanalysis/Vax_data.csv).
- Line 5 is a check to ensure the data was read correctly. The command asks for a list of the names of the variables in the newly created R data file, enabling you to ensure the file was read correctly. It is advisable to use R Studio so that you can click on the data file to see what it looks like. In R, the View function can be used to see the data: View(cfa_data).
- Line 8: All analyses in R are conducted with packages called libraries. Bifactor analysis was conducted with the package “lavaan,” and line 8 loaded this package. Packages must be installed before being used (R Studio does so with the option “Tools”).
- Lines 11–17 are the specifications of the bifactor model. The command created an object called model_bifactorVAX where the subscales of mistrust, future, profits, and natural were defined by three items each, and all 12 items of the VAX defined the total scale. Notably, the whole model is enclosed in single quotes (‘’), and the specification for the items that define a subscale is the equal sign (=) followed by the tilde (~). If copying and pasting from a Word document the single quote mark produced in Word is not the same as the single quote mark produced in R, even though it is the same keyboard stroke. Hence, replace the Word version of ‘with the R version of ’. In this regard it is preferable to use a text editor such as Notepad to compose your R Scripts as it produces the same characters as in R.
  Note that a bifactor model is seldom evaluated in isolation but usually compared to other models, such as one‐factor and correlated factor models. In R Script&amp;#x000A0;1, lines 12–15 represent the correlated (oblique) four‐factor model, while lines 16–17 represent the one‐factor model.

The next step was to fit the model to the data and examine the extent to which the model fit the data. The script is shown in R Script 2.

**R Script 2:**
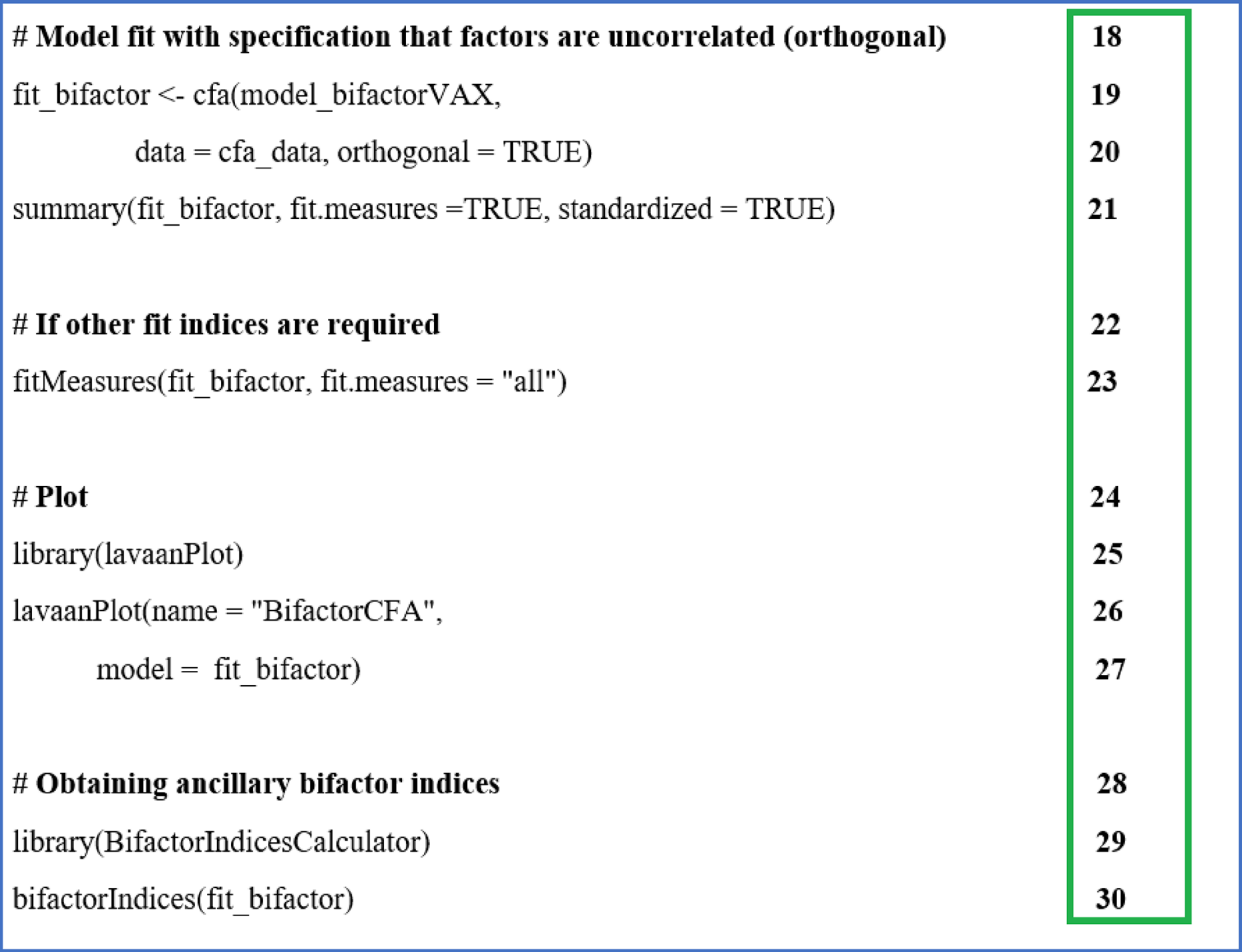
Fitting the bifactor model to the data and obtaining fit indices.

- Lines 19–20 created an object called “fit_bifactor” by specifying that CFA must be used to fit the model created in R Script 1 (model_bifactorVAX) to the data (cfa_data). A bifactor model assumes that the specific group factors are uncorrelated, specifying “orthogonal = TRUE.” This specification would not be required when examining a correlated (oblique) factor model.
- The summary function in line 21 specifies the output required, namely fit measures and standardised coefficients. The resultant output is shown in R Outputs 1 and 2.

**R Output 1:**
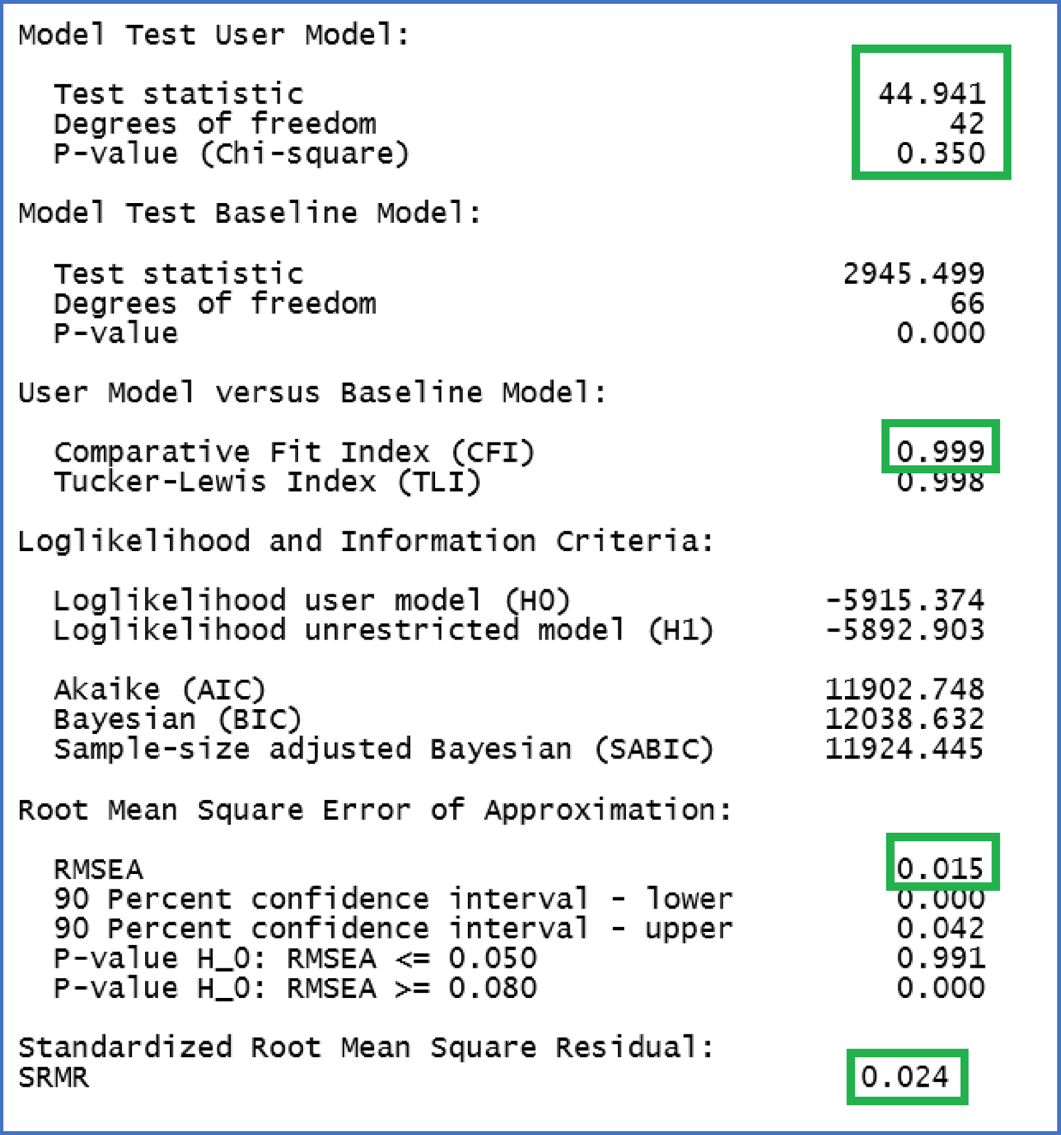
Default fit indices obtained with lavaan in R.

**R Output 2:**
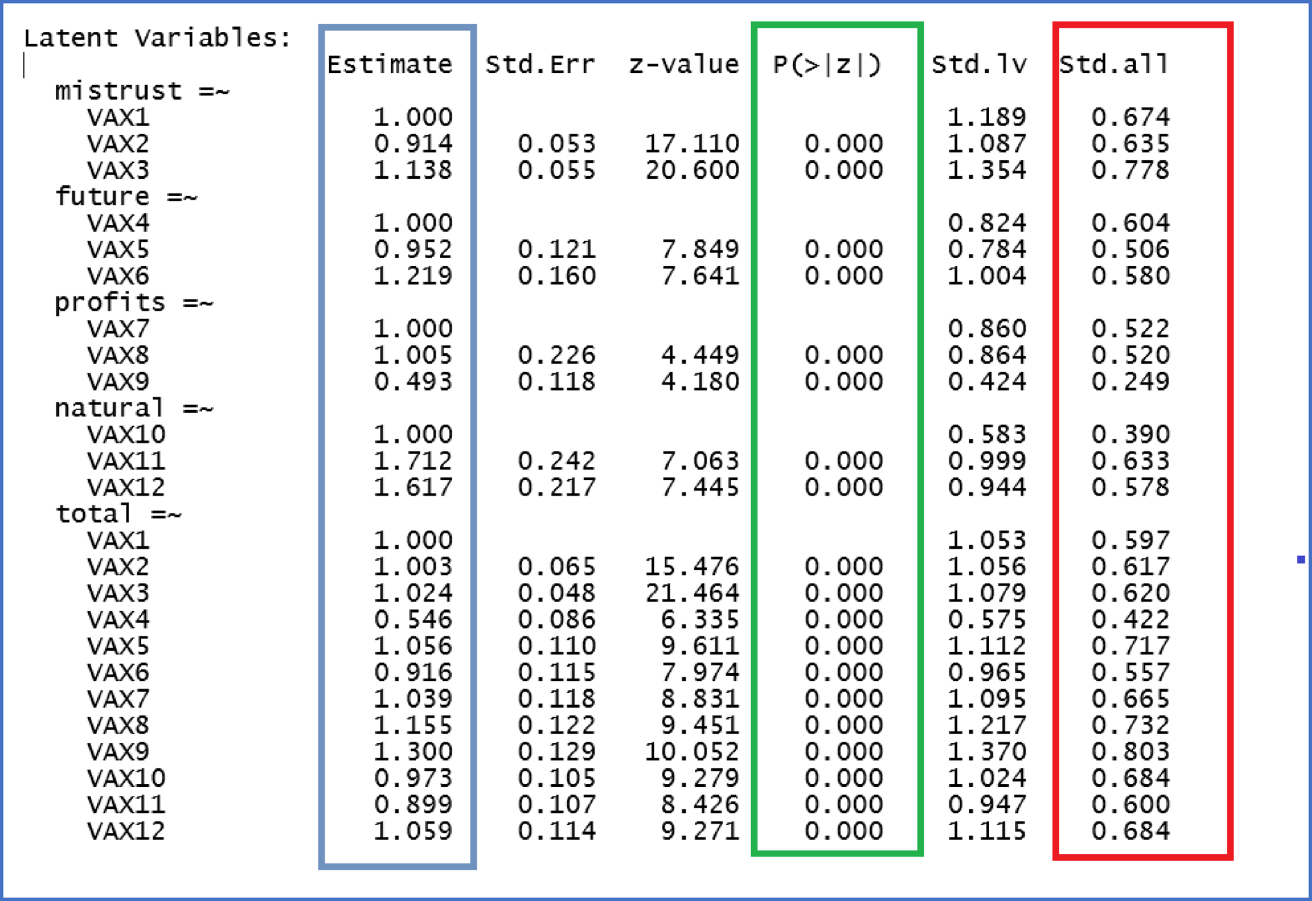
Standardised coefficients for the bifactor model of the vaccination attitude scale.

Above are the default fit indices provided by the summary specification in R Script 2. The relevant fit indices recommended by Kline (2015) are highlighted in green: χ^2^ = 44.94, *df* = 42, *p* > .05; CFI = .99; RMSEA = .02; SRMR .02. These indices demonstrated that the bifactor model of the VAX was almost a perfect fit for the data. The standardised coefficients requested in line 21 of R Script 2 are shown in R Output 2.

**Figure 3:**
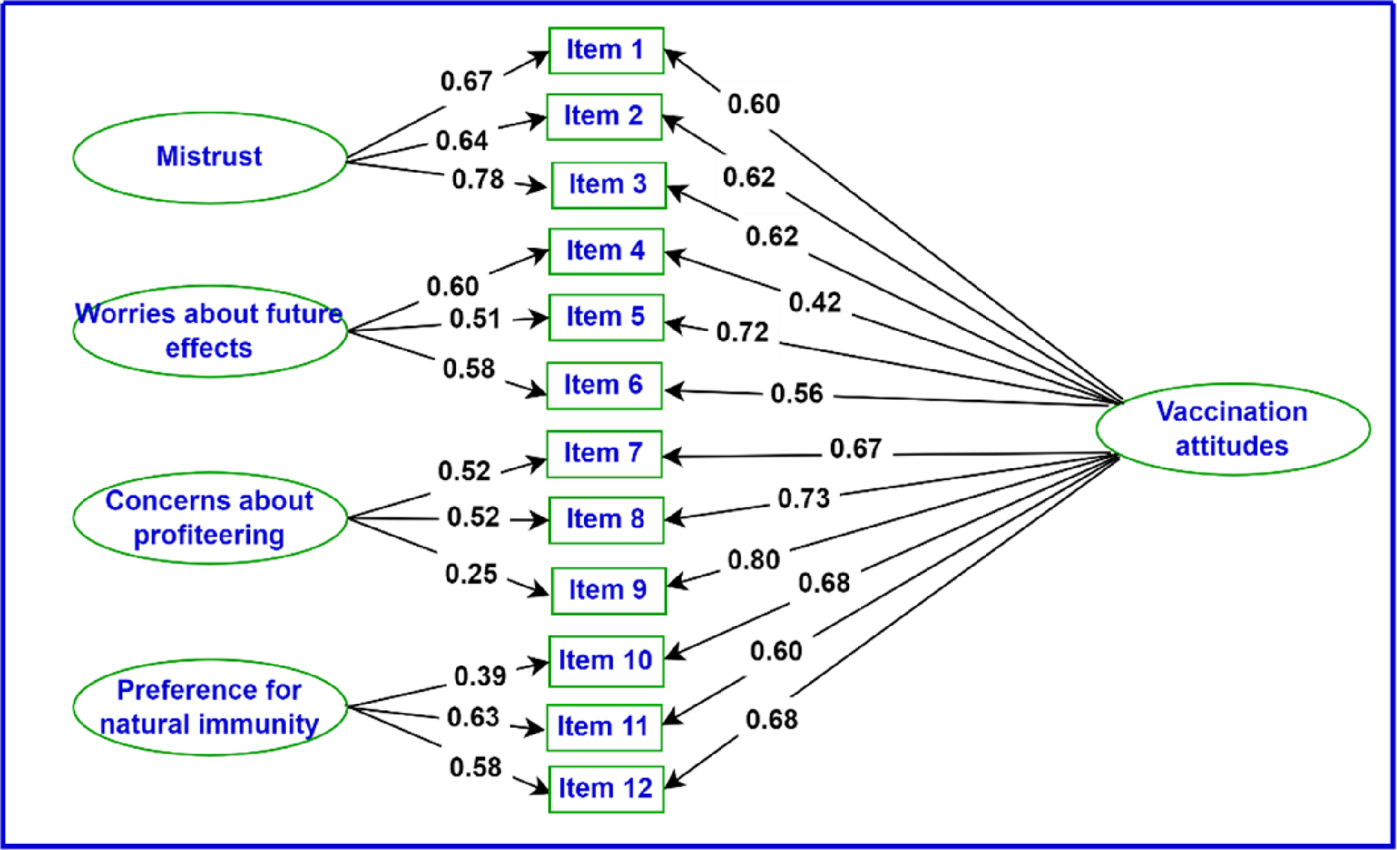
Bifactor model of the vaccination attitude scale with standardised regression coefficients. *Note*: Rectangles are observed variables; ellipses are latent variables.

The first column in R Output 2 lists the specific and general factors with the items loading on each. The blue column indicates unstandardised coefficients, the red column standardised coefficients, and the green column the significance of the coefficients. The bifactor model with standardised regression coefficients is presented in Figure 3.

The GFI was not included in the default output in R Output 1. Reviewers, supervisors, or instructors often suggest other indices for examination. Line 23 of R Script 2 can be used to obtain all the fit indices. Alternatively the function “fitmeasures()” can be used to obtain the indices you want: e.g., fitmeasures (fit_bifactor, c('gfi', 'nfi')).

The first column in R Output 2 indicated that the model was correctly specified and the correct items were loaded on their respective factors. This indication was double‐checked by requesting a plot of the model using lines 24–27 in R Script 2 to request a Lavaan plot.

Lastly, lines 29–30 in R Script 2 requested ancillary bifactor indices using the package “BifactorIndicesCalculator.” Note that the object created in line 19, “fit_bifactor,” was the model for which bifactor indices were requested. The output is shown in R Output 3.

**R Output 3:**
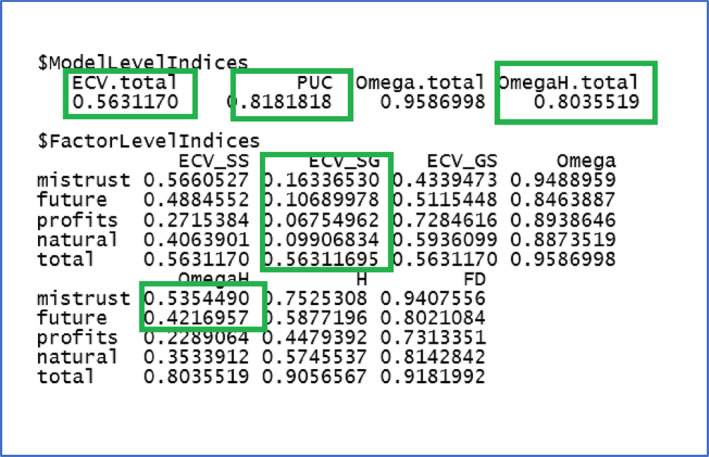
Ancillary bifactor indices for the vaccination attitude scale.

In R Output 3, ECV_SG is equivalent to Dueber's ECV. The bifactor indices suggested that the instrument had a strong general factor, as evidenced by the high OmegaH, PUC, H, and FD values. However, the ECV value of .56 also indicated some relevant specific group factors contributing to the common variance, as the specific group factors accounted for slightly less than half of the variance (43.7%). This indication was supported by the OmegaHS mistrust factor, which was above .50. In essence, while the instrument seemed to have a dominant general factor, it was not purely unidimensional, so specific group factors could provide valuable additional information.

## CONCLUSION

This tutorial sought to first demonstrate the importance of going beyond fit indices in bifactor models by examining ancillary bifactor indices to determine the proportion of variance explained by general and specific group factors. Secondly, the tutorial included a step‐by‐step guide to using R to examine model fit with CFA and obtain ancillary bifactor indices. By emphasising the critical role of ancillary indices such as the ECV, OmegaH and the PUC, researchers can be better equipped to make informed decisions about the dimensionality of psychological measurement instruments. Additionally, through practical examples and case studies, this tutorial underlined the potential limitations of relying solely on fit indices and advocated for a more nuanced and comprehensive approach to dimensionality assessment. Such an approach can ensure that the interpretation of factor structures is grounded in a thorough evaluation of both traditional fit measures and ancillary bifactor indices, ultimately enhancing the quality and accuracy of psychological measurement.

## COMPLIANCE WITH ETHICAL STANDARDS

The study was conducted according to the guidelines of the Declaration of Helsinki and approved by the Humanities and Social Science Research Ethics Committee of the University of the Western Cape (Ethics reference number: HS22/2/9, February 2022). All participants provided informed consent on the landing page of the electronic link.

## Endnotes

^1^
The results of the analyses for the two studies are reported in Pretorius, T.B. (2021). Overreliance on model fit indices in confirmatory factor analyses may lead to incorrect inferences about bifactor models: A cautionary note. *African Journal of Psychological Assessment*, *3*, Article 35. https://doi.org/10.4102/ajopa.v3i0.35.

^2^
The results of the analyses are reported in Padmanabhanunni, A., Pretorius, T. B., & Isaacs, S. A. (2023). Validation of the vaccination attitudes examination scale in a South African context in relation to the COVID‐19 vaccine: quantifying dimensionality with bifactor indices. *BMC Public Health*, *23(1)*, 1–8. https://doi.org/10.1186/s12889‐023‐16803‐4.

## APPENDIX A R SCRIPT FOR BIFACTOR ANALYSIS

**# Reading data with first row as header**
cfa_data <‐ read.csv("Vax_data.csv", header = T)
**# Check names of variables**
names(cfa_data)
**# Load appropriate library**
library(lavaan)
**# Specifying the bifactor model. Four specific factors and one general factor**
model_bifactorVAX <‐ '
mistrust =~ VAX1 + VAX2 + VAX3
future =~ VAX4 + VAX5 + VAX6
profits =~ VAX7 + VAX8 + VAX9
natural =~ VAX10 + VAX11 + VAX12
total =~ VAX1 + VAX2 + VAX3 + VAX4 + VAX5 + VAX6 + VAX7 + VAX8 + VAX9 + VAX10 + VAX11 + VAX12'
**# Model fit with specification that factors are uncorrelated (orthogonal)**
fit_bifactor <‐ cfa(model_bifactorVAX,
 data = cfa_data, orthogonal = TRUE)
summary(fit_bifactor, fit. measures =TRUE, standardized = TRUE)
**# If other fit indices are required**
fitMeasures(fit_bifactor, fit. measures = "all")
**# Plot**
library(lavaanPlot)
lavaanPlot(name = "BifactorCFA",
 model = fit_bifactor)
**# Obtaining ancillary bifactor indices**
library(BifactorIndicesCalculator)
bifactorIndices(fit_bifactor)

## APPENDIX B ITEMS OF THE VACCINATION ATTITUDE SCALE

**Response Scale:**

**Strongly disagree            Strongly agree.**

**1    2  3  4  5      6**

1. I feel safe after being vaccinated
2. I can rely on vaccines to stop serious infectious diseases
3. I feel protected after getting vaccinated
4. Although most vaccines appear to be safe, there may be problems that we have not yet discovered
5. Vaccines can cause unforeseen problems in children.
6. I worry about the unknown effects of vaccines in the future.
7. Vaccines make a lot of money for pharmaceutical companies, but do not do much for regular people
8. Authorities promote vaccination for financial gain, not for people's health
9. Vaccination programmes are a big con.
10. Natural immunity lasts longer than a vaccination
11. Natural exposure to viruses and germs gives the safest protection.
12. Being exposed to diseases naturally is safer for the immune system than being exposed through vaccination.
